# Mimicking 2,2′:6′,2′′:6′′,2′′′-quaterpyridine complexes for the light-driven hydrogen evolution reaction: synthesis, structural, thermal and physicochemical characterizations†

**DOI:** 10.1039/c9ra04303a

**Published:** 2019-09-06

**Authors:** Sanil Rajak, Olivier Schott, Prabhjyot Kaur, Thierry Maris, Garry S. Hanan, Adam Duong

**Affiliations:** Département de Chimie, Biochimie et Physique, Institut de Recherche sur L’Hydrogène, Université du Québec à Trois-Rivières Trois-Rivières Québec G9A 5H7 Canada adam.duong@uqtr.ca; Département de Chimie, Université de Montréal Montréal Québec H3C 3J7 Canada

## Abstract

The synthetic difficulties associated with quaterpyridine (qtpy) complexes have limited their use in the formation of various metallosupramolecular architectures in spite of their diverse structural and physicochemical properties. Providing a new facile synthetic route to the synthesis of functionalised qtpy mimics, we herein report the synthesis of three novel –NH_2_ functionalized qtpy-like complexes 12–14 with the general formula M(C_16_H_14_N_12_)(NO_3_)_2_ (M = Co(ii), Ni(ii) and Cu(ii)) in high yield and purity. Characterization of these complexes has been done by single crystal X-ray diffraction (SCXRD), thermogravimetric analysis, UV-Vis, infrared, mass spectrometry and cyclic voltammetry. As indicated by SCXRD, in all the synthesized complexes, the metal ions show a strongly distorted octahedral coordination geometry and typical hydrogen bonding networks involving DAT groups. In addition, complexes 12–14 have been analyzed as potential photocatalysts for hydrogen evolution reaction (HER) displaying good turnover numbers (TONs). Hydrogen produced from these photocatalysts can serve as the possible alternative for fossil fuels. To the best of our knowledge, this is the only study showcasing –NH_2_ functionalized qtpy-like complexes of Co(ii), Ni(ii) and Cu(ii) and employing them as photocatalysts for HER. Thus, a single proposed strategy solves two purposes-one related to synthesis while second is related to our environment.

## Abstract 2

Facile synthesis of three novel –NH_2_ functionalized qtpy-like complexes, their characterizations and study of their photocatalytic properties for hydrogen evolution reaction.

## Graphical abstract

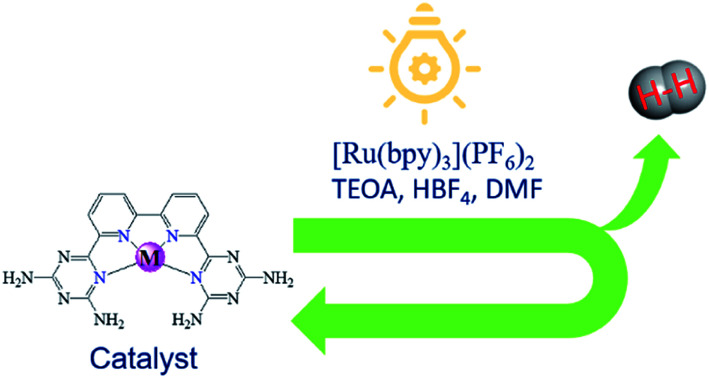

## Introduction

Complexes based on coordination of both unsubstituted and functionalized bipyridine (bpy), terpyridine (tpy) and quaterpyridine (qtpy) with transition metals have been widely studied in particular for the formation of various metallosupramolecular architectures.^1–8^ There is very extensive literature concerning polypyridine ligands and their complexes, but a relatively few investigations have been done on functionalized systems, particularly on qtpy. Many interests were focused on qtpy, a tetradentate ligand that can form metal complexes of different geometries. qtpy presents diverse features such as (i) *N*-heterocyclic scaffolds, (ii) predictable coordination chemistry, (iii) better oxidation resistance as compared to bi-and terpyridine and (iv) a low energy orbital for metal-to-ligand charge transfer transition in the visible region. Although evaluation of their structural and physicochemical properties drew considerable attention, their use has rapidly declined because of the synthetic difficulties which limit the prospects of their application.

**Chart 1:**
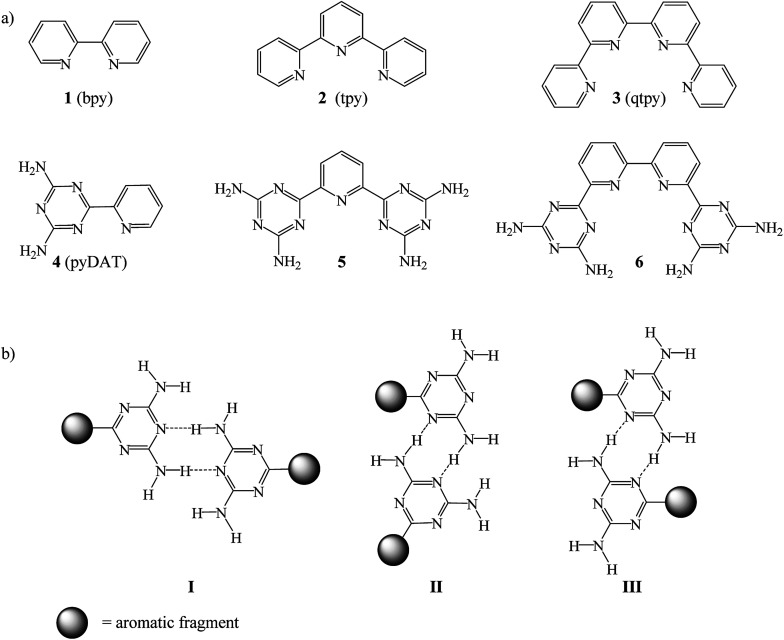
Molecular structures of (a) compounds 1–6 and (b) the hydrogen bonds motifs of DAT groups.

Therefore, an alternative synthetic approach that consists of replacing one or several pyridine rings by a diamino-1,3,5-triazinyl group (DAT) has been employed to facilitate easy preparation of bpy, tpy and qtpy-type ligands 1–3.^9^ Compounds 4–6 were designed to eliminate the most serious drawbacks concerning functionalization of bpy, tpy and qtpy synthesis (Chart 1a). These compounds are pyridyl and bipyridyl substituted in *ortho* with one or two DAT groups. In crystal engineering these molecules are known as tectoligands because of their dual ability to bind metal ions and simultaneously engage in predictable intermolecular interactions such as hydrogen bonds according to reliable patterns (Chart 1b).^5,10,11^ In supramolecular chemistry, the self-assembly of tectoligands with metal ions forms metallotectons.^12–18^ Creation of metallosupramolecules^19,20^ using 4–6 has several advantages: (i) the synthesis is easy and the yield is high, (ii) the coordination chemistry is similar to 1–3, (iii) in solid-state, reliable hydrogen bond networks with predefined structures are expected, (iv) the presence of amino groups and triazinyl rings may lead to fine tuning of activities of the metal complexes for catalysis.

**Chart 2:**
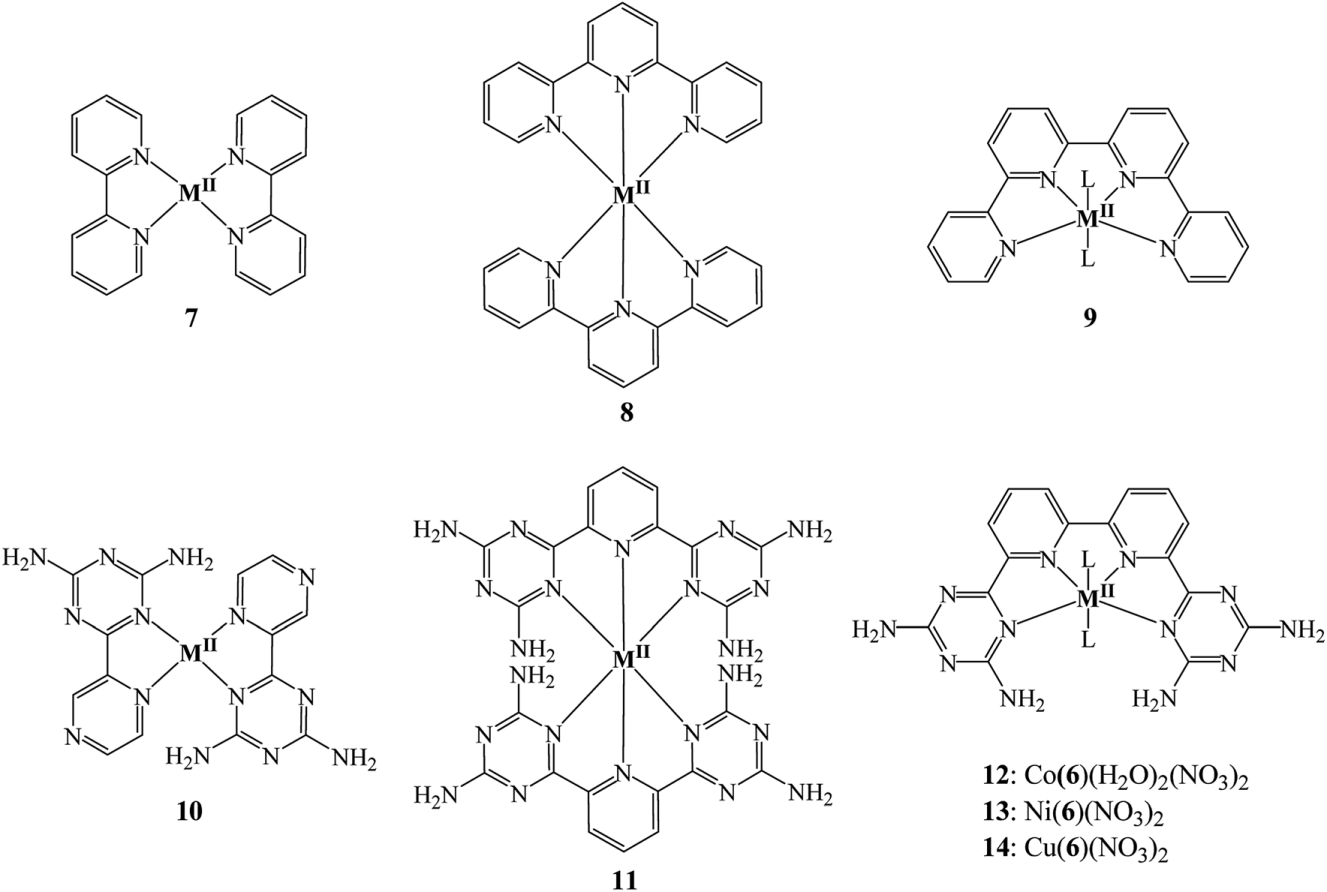
Molecular structures of complexes 7–14.

To the best of our knowledge, coordination chemistry of 6 has never been investigated. Compound 6 is expected to function as a tetradentate ligand chelating ions to form helical metallotectons 12–14 (Chart 2). The range of applications of complexes of 6 may include molecular machines, supramolecular functional devices, catalysts for both organic and inorganic reactions, biomedical like DNA binding, medicinal chemistry, nonlinear optical materials and so forth.^21–27^

In this present study, our interests focus on the coordination chemistry of 6 to form complexes 12–14 and their self-assembly by hydrogen bonds *via* the DAT groups. This work is an attempt to provide a remedy for the lack of straightforward and efficient synthetic pathways to prepare functionalized 2,2′:6′,2′′:6′′,2′′′-quaterpyridine (qtpy) complexes by minimizing the steps involved in their synthesis and obtain these complexes in high yield and purity without the need for any further purification. We have chosen first row transition metals to form complexes with 6 because of their low cost compared to platinum group metals. Another aspect of the present work is to provide a possible future alternative for fossil fuels in the form of a clean fuel – hydrogen which can be generated by a renewable energy source – sunlight so as to protect our environment from greenhouse effect and global warming and to meet the ever increasing energy requirements.^28–31^ With the depletion of fossil fuels, considerable efforts have been made by chemists to convert solar energy into storable chemical forms. Currently, molecular hydrogen (H_2_) is one of the most promising sustainable energy supplies to replace conventional gasoline and diesel fuel because its combustion produces high energy density and non-toxic emissions. However, hydrogen is not readily available in the atmosphere. It is mainly produced by electrolysis and steam reforming which are not economically viable and environmentally polluting, respectively. The search for efficient and cost-effective methods to produce H_2_ is therefore one of the most challenging tasks for the next few decades. A sunlight-triggered hydrogen evolution reaction (HER) would be an interesting solution. Although, catalysts for HER have been the subject of several reviews, considerable efforts are needed to provide an effective method to convert solar energy into hydrogen.^32–37^ Since complexes 12–14 are similar to qtpy complexes which are known to show diverse catalytic activities, they should be of particular utility in major contemporary fields such as solar energy conversion. In addition, the amino groups present in DAT group can further enhance the photocatalytic activity of complexes.^38,39^ Therefore, we have also tested 12–14 for hydrogen evolution reaction (HER).

## Results and discussion

### Syntheses and characterizations

6,6′-(2,2′-Bipyridine-6,6′-diyl)bis(1,3,5-triazine-2,4-diamine) 6 was obtained by reported method.^40^ Complexes 12–14 were prepared by mixing the ligand (1 equiv.) with M(NO_3_)_2_·*x*H_2_O (1 equiv.) in MeOH. The precipitated solids were dissolved in DMSO and crystallized by slow diffusion with diethyl ether or ethyl acetate. Electrospray mass spectrometry of 12–14 gave peaks at *m*/*z* = 495.06, *m*/*z* = 216.04 and *m*/*z* = 218.54 which were assigned to species [Co(6)(NO_3_)]^+^, [Ni(6)]^2+^ and [Cu(6)]^2+^, respectively. The infrared spectra of these products showed the presence of NO_3_^−^ group observed at 1313, 1313, 1323 cm^−1^ for complexes 12, 13 and 14, respectively (Fig. S5†). The existence of ligand 6 was confirmed with –NH_2_ bands in the range 3150–3500 cm^−1^. The band positions for each spectrum are summarized in Table S4† along with their proposed assignments. The compositions found by elemental analysis (EA) for each sample gives the general chemical formula M(C_16_H_14_N_12_)(NO_3_)_2_ (M = Co(ii), Ni(ii) and Cu(ii)) which is consistent with the expected structures (see Experimental section).

### Crystal structures of 12–14

In accordance with the previous investigation on qtpy ligands and their complexation by transition metal ions which demonstrate that 2,2′:6′,2′′:6′′,2′′′-quaterpyridine tend to form mainly mononuclear complexes with metal ions favouring square planar or octahedral coordination geometry; the crystal structures of our complexes determined by SCXRD also showed that the ligand adopts a planar conformation with all N-donor sites oriented internally and the geometry of the resultant complexes were found to be distorted octahedral. However, these features can be modified upon the particular substitution pattern added to the oligopyridine core. The coordination of ligand 6 with cobalt(ii), nickel(ii) and copper(ii) metal ions are expected to produce six-coordinate metal complexes in which the ligand has to twist out of planarity owing to steric interaction of the NH_2_ of DAT groups. In all structures that we reported here, the metal ions show a strongly distorted octahedral coordination geometry and typical hydrogen bonding networks involving DAT groups (Chart 1b).

**Fig. 1:**
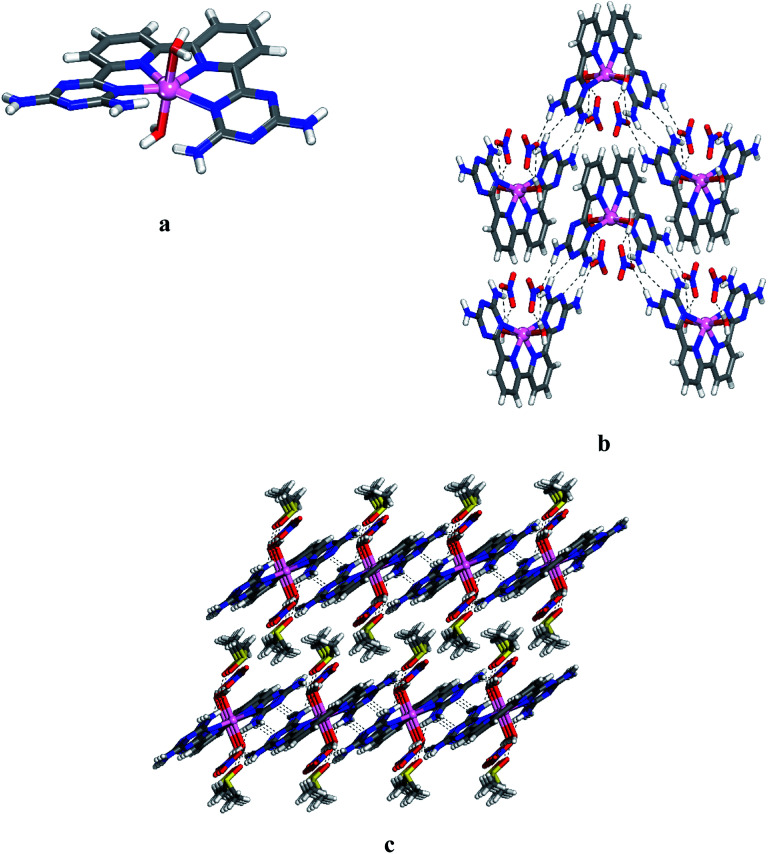
Crystal structure of 6,6′-(2,2′-bipyridine-6,6′-diyl)bis(1,3,5-triazine-2,4-diamine)(diaqua)cobalt(ii) nitrate 12 grown from DMSO/EtOAc. (a) Perspective view of one cation of 12, [Co(6)(H_2_O)_2_]^2+^. (b) Alternating arrangement of complex 12 and its enantiomer to form chains mainly by hydrogen bonds involving DAT groups. Chains are then joined by π–π stacking of heteroaromatic rings. (c) Alternating packing of layers of complexes and layers of DMSO. Hydrogen bonds are represented by dashed lines. Unless stated otherwise, carbon atoms are shown in gray, hydrogen atoms in white, oxygen atoms in red, nitrogen atoms in blue and cobalt atoms in pink.

Crystals of 12 grown from DMSO/EtOAc were found to belong to the monoclinic space group *C*2/*c*. Views of the structure are shown in Fig. 1, and other crystallographic data are provided in Table 1. The central cobalt atom adopts the common coordination geometry for six-coordinated Co(ii) with 2,2′:6′,2′′:6′′,2′′′-quaterpyridine ligand type (Fig. 1a). The equatorial sites are occupied by the four inter nitrogen atoms of compound 6. The two central Co–N_py_ bonds are slightly shorter than the two outer Co–N_DAT_ bonds. This is due to the constrained effect of the qtpy ligand type. Two water molecules are in axial positions to complete the coordination sphere to form the cationic complex [Co(6)(H_2_O)_2_]^2+^. The distance Co–N and Co–O within the complex (2.173 Å for Co–N_DAT_, 2.085 Å for Co–N_py_ and 2.124 Å for Co–O) have normal values in comparison with reported structure of [Co(qtpy)(H_2_O)](NO_3_)_2_.^41^ The helicity of the ligand 6 in the complex is due to the steric interaction between the two NH_2_ of DAT groups (Fig. 1a). In the crystal, both chiralities are observed and they are joined alternately into chains by characteristic hydrogen bonding of type I between DAT groups (average N–H⋯N distance = 3.059 Å), strengthened by additional N–H⋯O hydrogen bonds involving bridging of nitrate (Fig. 1b). With the assistance of hydrogen bonds involving bridging of DMSO and π–π stacking of heteroaromatic rings, the cationic chains pack to form layers, and the layers stack to produce the observed three-dimensional structure (Fig. 1c). Details of the hydrogen bonds and their angles are summarized in Table S1.†

**Table 1:** Crystallographic data for complexes 12–14^a^

	12	13	14
Crystallization condition	DMSO/EtOAc	DMSO/EtOAc	DMSO/THF
Empirical formula	C_10_H_15_CoN_7_O_5_S	C_32_H_32_N_28_Ni_2_O_14_ + solvent	C_18_H_18_CuN_14_O_7_ + solvent
Formula weight	374.81	1150.27^a^	606.0^a^
Crystal system	Monoclinic	Triclinic	Monoclinic
Radiation	GaKα	GaKα	GaKα
Temperature (K)	150	150	150
*λ* (Å)	1.34139	1.34139	1.34139
*F*(000)	1548	588^a^	1236^a^
Space group	*C*2/*c*	*P*1̄	*P*2_1_/*n*
*Z*	8	1	4
*a* (Å)	24.2963(6)	9.6118(5)	10.1245(3)
*b* (Å)	10.0731(3)	11.7609(6)	15.8382(4)
*c* (Å)	13.8796(4)	14.1181(8)	21.6268(6)
*α* (deg)	90	95.139(3)	90
*β* (deg)	111.106(10)	91.020(3)	102.018(1)
*γ* (deg)	90	113.356(2)	90
*V* (Å^3^)	3169.00(15)	1456.81(14)	3391.93(16)
Crystal size/mm^3^	0.12 × 0.07 × 0.06	0.15 × 0.06 × 0.06	0.17 × 0.12 × 0.1
*ρ*_calcd_ (g cm^−3^)	1.571	1.311^a^	1.187^a^
*μ* (mm^−1^)	4.163	3.961^a^	3.772^a^
Reflections collected	24 490	21 341	49 606
Independent reflections	3634	6685	7797
*R*_int_	0.0536	0.0583	0.0453
Observed reflections	3113	5349	7080
2*θ* range for data collection/°	6.79 to 121.48	5.46 to 121.67	6.06 to 121.65
Data/restraints/parameters	3634/0/217	6685/0/345	7797/4/393
*R*_1_ [*I* > 2*σ*(*I*)]	0.0426	0.0554	0.0677
w*R*_2_ [*I* > 2*σ*(*I*)]	0.1057	0.1483	0.2067
*R*_1_ (all data)	0.0531	0.0689	0.0721
w*R*_2_ (all data)	0.1122	0.1569	0.2110
Goodness-of-fit on *F*^2^	1.026	1.045	1.105
Largest diff. peak/hole/e Å^−3^	0.60/−0.48	0.562/−0.514	0.933/−0.583

a Disordered solvent molecules that were treated by a mask/squeeze procedure are not included in the calculation.

**Fig. 2:**
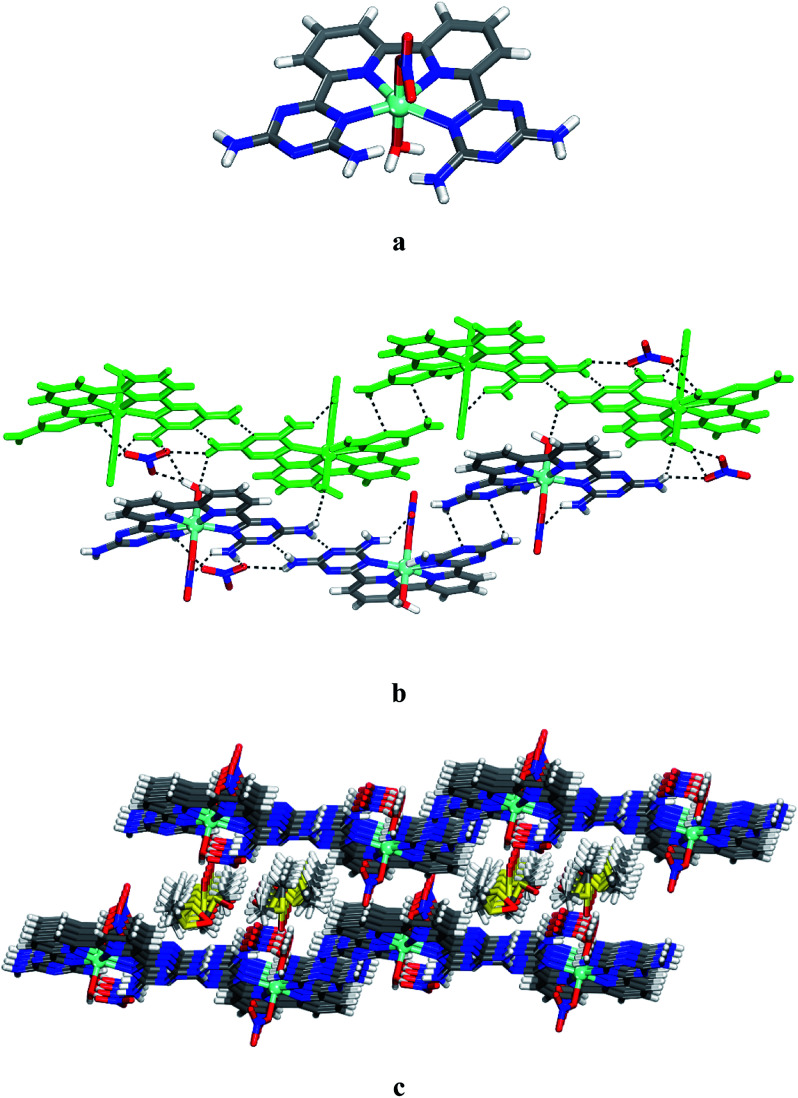
Views of crystal structure of the 6,6′-(2,2′-bipyridine-6,6′-diyl)bis(1,3,5-triazine-2,4-diamine)(aqua)(nitrato-O)nickel(ii) 13 grown from DMSO/THF. (a) Perspective view of a cation of 13, [Ni(6)(NO_3_)(H_2_O)]^+^. (b) Alternating arrangement of complex 13 and its enantiomer held together by hydrogen bonds *via* DAT groups according to motif I and by bridging involving nitrate counter ion. For clarity one chain is marked in green. (c) View of alternating layers of complexes and disordered DMSO. Hydrogen bonds are represented by dashed lines. Unless stated otherwise, carbon atoms are shown in gray, hydrogen atoms in white, oxygen atoms in red, nitrogen atoms in blue and nickel atoms in cyan.

To further assess the potential of 6 to form various complexes with transition metals, we examined the product of the reaction of this ligand with Ni(NO_3_)_2_·6H_2_O. Crystals of 13 grown from DMSO/EtOAc were found to have the composition [Ni(6)(NO_3_)(H_2_O)]·(NO_3_)·4DMSO and belonged to the triclinic space group *P*1̄. Views of the structure are presented in Fig. 2, and other crystallographic data are provided in Table 1. In the structure, ligand 6, a nitrate and a water molecule bind the metal ion to form a cationic complex [Ni(6)(NO_3_)(H_2_O)]^+^ (Fig. 2a). The coordination geometry of the nickel can be considered as a distorted octahedral. The distance Ni–N and Ni–O within the complex (average distances = 2.166 Å for Ni–N_DAT_, 2.021 Å for Ni–N_py_, 2.083 Å for Ni–O_water_ and 2.071 Å for Ni–O_nitrate_) are consistent with those reported for quaterpyridine nickel complexes.^42^ A direct comparison between the two structures with that of [Co(6)(H_2_O)_2_]^2+^ and [Ni(6)(NO_3_)(H_2_O)]^+^ reveals that the helicity is less marked in nickel complex 13. As with 12, the crystal structure of 13 is a racemate. The complex of 13 and its enantiomers are joined by hydrogen bonds of DAT groups according to motif I (average distance N–H⋯N = 2.462 Å) and by bridging of nitrate (N–H⋯O = 2.177 Å) to form zigzag chains. The chains are linked by hydrogen bonds involving oxygen atom from water molecule and the free hydrogen atom of –NH_2_ group, and by the bridging of NO_3_^−^ to produce layers (Fig. 2b). The final structure consists of alternating layers of complexes and molecules of DMSO (Fig. 2c). It is noteworthy that the geometry of coordination of the metal ion is reinforced by the intramolecular hydrogen bonds involving nitrato ligand and one of the –NH_2_ groups. Selected hydrogen bonds and their angles are given in Table S2.†

**Fig. 3:**
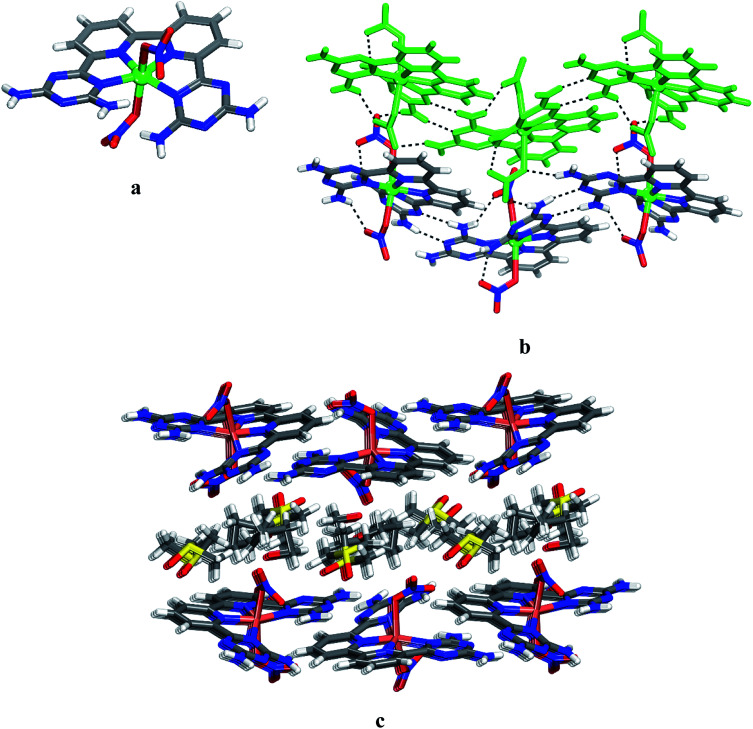
Crystal structure of 6,6′-(2,2′-bipyridine-6,6′-diyl)bis(1,3,5-triazine-2,4-diamine)(nitrato-O)copper(ii) 6 grown from DMSO/THF. (a) Perspective view of 14, Cu(6)(NO_3_)_2_. (b) Alternating arrangement of complex 14 and its enantiomer, which are held together by hydrogen bonds *via* DAT groups according to motif II and by bridging involving nitrato ligand and the free hydrogen of –NH_2_ group. For clarity one chain is marked in green. (c) View of alternating layers of complexes and disordered DMSO. Hydrogen bonds are represented by dashed lines. Unless stated otherwise, carbon atoms are shown in gray, hydrogen atoms in white, oxygen atoms in red, nitrogen atoms in blue and copper atoms in green.

Unsurprisingly, the structure of Cu^II^ complex 14 closely resembles that of 12 and 13. Views of the structure are shown in Fig. 3, and other crystallographic data are given in Table 1. The coordination geometry of the copper atom can be described as a distorted octahedral with the four N inter atoms in equatorial and the two nitrates in axial positions. The average distances Cu–N_py_ and Cu–N_DAT_ are 1.962 Å and 2.065 Å, respectively (Fig. 3a). These values are consistent with those reported for quaterpyridine copper complexes.^41^ The measured distances of the two Cu–O bonds (2.569 Å and 2.283 Å) suggest a Jahn–Teller effect.^43,44^ In 14, the N_DAT_–Cu–N_DAT_ void angle of 125.3(4)^o^ is somewhat less than the related value of 135.8(9)^o^ in 12 and 129.4(1)^o^ in 13. In the structure of 14, each DAT group is linked by two N–H⋯N hydrogen bonds of type II (average distance N–H⋯N = 3.075 Å) to form chains. Chains are then joined together *via* hydrogen bonds involving bridging of nitrato ligands to produce layers (Fig. 3b). Alternating packing of layers of complexes and DMSO generates the three-dimensional structure (Fig. 3c). Details of the hydrogen bonds and their angles are provided in Table S3.†

### Thermal analysis of 6 and 12–14

Thermogravimetric analysis (TGA) were recorded on compounds 6 and 12–14 (Fig. S4†). The samples were heated from *ca.* 25 to 800 °C at a rate of 10 °C min^−1^ under nitrogen atmosphere. The TG curve of the free ligand 6 present three steps (Fig. S4a†). The first variation of mass at 117 °C is attributed to the loss of water molecules that are hydrogen bonded with the DAT groups. Decomposition of 6 starts at 434 °C. Compound 12 displays three steps mass losses (Fig. S4b†). The first slight inflection at 100 °C with a mass loss of ∼5% is indicative of the loss of approximately two water molecules. The second and third steps in the range 307–367 °C and 367–662 °C present mass losses of ∼28% and ∼25%, respectively. TG curves of 13 and 14 show similar patterns with the first decomposition that starts at ∼270 °C (Fig. S4c and d†).

### Catalytic properties

#### Electronic characterization

**Fig. 4:**
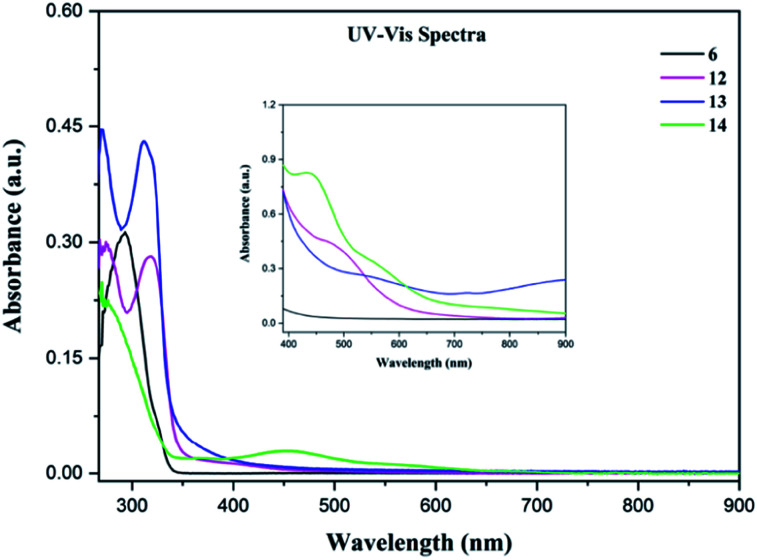
UV-Vis spectra of 6 and 12–14 in DMF solution at room temperature at a concentration of 8.8 × 10^−6^ M. Inset shows the visible region (390–900 nm) at a concentration 8 × 10^−3^ M.

UV-Vis absorption spectra of 6 and 12–14 were performed at room temperature in DMF solution at concentration 8.8 × 10^−6^ M and at 8 × 10^−3^ M (Fig. 4). The electronic absorption spectrum of the free ligand 6 shows an intense absorption band at 292 nm accompanied with a small shoulder at 325 nm which is attributed to *n*–π* and π–π* transitions. The UV-Vis absorption spectra of 12 and 13 are similar. There are two intense intraligand transitions and a weak inflection between 350–500 nm that can be attributed to the d–d transitions.^45^ In the case of 14, one large band at around 275 nm is observed in the UV region and is assigned to intraligand transitions. Also, a broad band at 450 nm can be assigned to MLCT (Metal Ligand Charge Transfer) electronic transition.^46,47^ Selected data (wavelengths (*λ*_max_), molar absorptivity (*ε*)) are summarized in Table 2.

**Table 2:** Liquid state UV-Vis data of 6 and 12–14^a^

Sample	Parameters (in DMF solution)			
	Conc = 8.8 × 10^−6^ M from 267 to 900 nm		Conc = 8 × 10^−3^ M from 390 to 900 nm	
	*λ*_max_ (nm)	*ε* (mol^−1^ dm^3^ cm^−1^)	*λ*_max_ (nm)	*ε* (mol^−1^ dm^3^ cm^−1^)
6	292	3.56 × 10^4^		
	325	8.44 × 10^3^		
12	275	3.32 × 10^4^	470	5.62 × 10^2^
	318	3.19 × 10^4^		
	400	1.45 × 10^3^		
13	270	5.28 × 10^4^	546	3.2 × 10^2^
	311	4.92 × 10^4^	722	2.06 × 10^2^
	319	4.61 × 10^4^		
14	275	2.52 × 10^4^	435	1.03 × 10^3^
	371	2.21 × 10^3^	559	4.08 × 10^2^
	455	3.64 × 10^3^		
	555	1.38 × 10^3^		

a *λ*: wavelength (nm); *A*: absorbance and *ε*: molar absorptivity's (mol^−1^ dm^3^ cm^−1^).

**Fig. 5:**
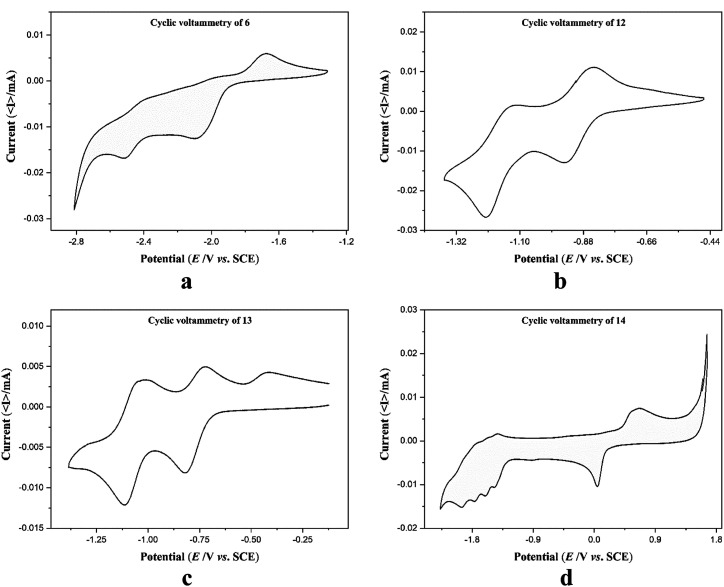
Cyclic voltammograms of ligand 6 and complexes 12–14.

Electrochemical measurements of the free ligand 6 and complexes 12–14 were performed in anhydrous and degassed DMF solutions at concentration of compound 1 mmol dm^−3^ with 0.1 M TBA-PF_6_ (tetrabutylammonium hexafluorophosphate) as a supporting electrolyte at a scan rate of 100 mV s^−1^. Cyclic voltammograms (CV) of 6 did not show any reversible redox waves in the range −2.9 to −1.1 V (Fig. 5a). CV of complexes 12 and 13 shows clearly quasi reversible redox peaks at −1.18 V and −0.90 V and −1.08 V and −0.78 V, respectively which are attributed to the diverse redox states of the cobalt and nickel metal ions (Fig. 5b and c). These values are comparable with those of quaterpyridine complexes reported in literature (Table S5†).^48,49^ In the CV of complex 14, there are multiple irreversible cathodic peaks in the negative range that correspond to the ligand reduction processes in 14 (Fig. 5d). The first redox event assimilated as the reduction of Cu(ii) to Cu(i) occurs at 0.04 V, while the redox event occurring at 0.64 V is due to the oxidation of Cu(i) to Cu(ii). The irreversible character of both linked events separated by 600 mV are presumably representative of rearrangement of coordination sphere or dimerization.^50^Table 3 summarizes the redox data of 6 and 12–14.

**Table 3:** Cyclic voltammetry data of 6 and 12–14 in DMF solution

Compound	*E* ^ox^_1_ [V]	*E* ^ox^_2_ [V]	*E* ^red1^_1/2_ [V]	*E* ^red2^_1/2_ [V]	*E* ^red3^_1/2_ [V]	*E* ^red4^_1/2_ [V]	*E* ^red5^_1/2_ [V]
6			−1.98(33)	−2.47(82)			
12			−0.90(15)	−1.18(09)	−1.47(02)	−1.78(22)	−2.19(49)
13			−0.78(43)	−1.08(29)	−1.63(45)	−1.94(48)	−2.25(50)
14	0.04(52)^a^	0.67(00)^a^	−1.43(85)^a^	−1.56(74)	−1.73(79)	−1.90(84)	

a Non-reversible.

### Hydrogen evolution reaction

The molecular structures of complexes 12–14 determined by single-crystal X-ray diffraction confirm that they are similar to mononuclear qtpy complexes. Previously, qtpy complexes have been used as photo/electro catalysts for CO_2_ reduction.^48,50,51^ As cobalt, nickel and copper complexes of 2,2′:6′,2′′:6′′,2′′′-quaterpyridine are known to be active catalysts for many chemical conversions, we have investigated the catalytic properties of complexes 12–14 for HER. To the best of our knowledge, there have been only two reports so far wherein non-functionalized qtpy complexes have been used for photocatalytic hydrogen production. In 2012, Leung and co-researchers synthesized [Co^II^(qtpy)(H_2_O)_2_]^2+^ complex which acted as an efficient photocatalyst for hydrogen generation from [Ir^III^(dF(CF_3_)ppy)_2_(dtbbpy)]^+^/TEOA (dF(CF_3_)ppy = anion of 2-(2,4-difluorophenyl)-5-trifluoromethylpyridine, dtbbpy = 4,4′-di-*tert*-butyl-2,2′- bipyridine, TEOA = triethanolamine) in aqueous acetonitrile giving a maximum TON of 1730 at a 420 nm wavelength.^52^ Recently, Hanan *et al.* synthesized [Ru(qpy)_3_]^2+^ (qpy = 4,4′:2′,2′′:4′′,4′′′-quaterpyridine) complex and found it to be a suitable photosensitizer for hydrogen evolution in red light from triethanolamine (TEOA), HBF_4_ (48% in water) and [Co(dmgH)_2_]^2+^ catalyst exhibiting a TON of 375 far greater than the TON of 30 of the most studied photosensitizer [Ru(bpy)_3_]^2+^.^53^ To the best of our knowledge, this is the first report wherein functionalised qtpy complexes have been used as potential photocatalysts for HER.

**Fig. 6:**
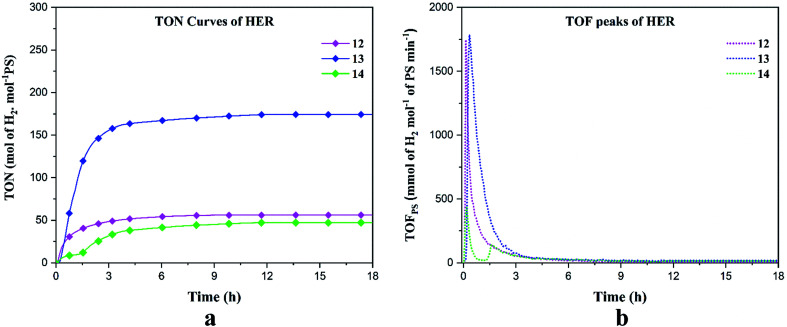
Hydrogen evolution reaction of 1 mM of 12–14 in blue light. (a) TON's and (b) TOF's.

We performed HER under blue light (452 nm) in DMF solution containing the catalysts 12–14, triethanolamine (TEOA) as the sacrificial electron donor, Ru(bpy)_3_(PF_6_)_2_ as the photosensitizer (PS) and aqueous HBF_4_ as the proton source. The experiment was conducted for 18 hours. The hydrogen production rate, turnover number (TON) and turnover frequency (TOF) have been reported in millimoles of hydrogen per hour, moles of hydrogen per moles of PS and mmol of hydrogen per mole of PS per minute respectively (Table 4). Under blue irradiation, the production of H_2_ starts almost instantaneously after turning the light on (Fig. 6). Control experiments were conducted in the presence of PS/TEOA alone with and without light and no H_2_ production was recorded which was consistent with the previous results.^54^ For all our complexes, the maximum hydrogen production was reached after ∼ 3 h and stayed constant for up to 18 h. The maximum turnover numbers (TON's) are 56, 174 and 47 mol_H_2__ mol_PS_^−1^ for 12–14, respectively (Fig. 6a). The turnover frequencies (TOF's) reached 1741 and 1782 mmol_H_2__ mol_PS_^−1^ min^−1^ for 12 and 13, respectively. In the case of complex 14, the TOF's are 430 and 137 mmol_H_2__ mol_PS_^−1^ min^−1^ (Fig. 6b). Complex 13 displayed superior HER properties because, the first reduction potential of 13 (*E*^red^_1/2_ = −0.78 V) is 120 mV less negative as compared to 12 (*E*^red^_1/2_ = −0.90 V) and since for 14, no reversible event was observed, this indicates that the excited Ru(ii) complex can more readily transfer electrons to 13 as compared to 12 and 14. To confirm the photocatalytic activity of our complexes, blank experiments were carried out with Co(NO_3_)_2_·6H_2_O, Ni(NO_3_)_2_·6H_2_O and Cu(NO_3_)_2_·2.5H_2_O which displayed TON's of 9.96, 18.54 and 0.58 mol_H_2__ mol_PS_^−1^ respectively, clearly indicating that the salt alone cannot act as a photocatalyst (Fig. S8†). In accordance with the results obtained by Probst *et al.* in 2009 who studied the effect of pH on the photocatalytic HER using the reference catalyst [Co(dmgH)_2_], we deduce that in our photocatalytic experiments, HBF_4_ is the major proton source, while water and TEOA acts as a subsidiary proton source.^55^ The mechanism of hydrogen evolution reaction can occur by the process of oxidative quenching of the excited Ru(ii)* complex proceeded by hydrogen production *via* heterolytic pathway, which is proposed in the ESI.†^56^

**Table 4:** Turnover number and turnover frequency maximal for complexes 12–14^a^

Compound	Molecular formula	H_2_ production (mmol h^−1^)	TON_max_	TOF_max_ (min^−1^)
12	Co(6)(NO_3_)_2_	0.31	56	1741
13	Ni(6)(NO_3_)_2_	0.96	174	1782
14	Cu(6)(NO_3_)_2_	0.26	47	430, 137

a TON is reported in moles of hydrogen per mole of PS and TOF in mmol of hydrogen per mole of PS per minute.

## Conclusion

In this work, the synthesis and characterisation of –NH_2_ functionalized qtpy-like complexes of Co(ii), Ni(ii) and Cu(ii) is undertaken and they are employed as photocatalysts for HER. Three novel –NH_2_ functionalized qtpy complexes 12–14 with the general formula M(C_16_H_14_N_12_)(NO_3_)_2_ (M = Co^II^, Ni^II^ or Cu^II^) have been successfully synthesized *via* an easy synthetic procedure giving high yield and purity. In all complexes, the metal ions show a strongly distorted octahedral coordination geometry and typical hydrogen bonding networks involving DAT groups. In addition, we investigated the photocatalytic activity of complexes 12–14 in DMF solution for HER under blue light (452 nm) using triethanolamine (TEOA) as the sacrificial electron donor, Ru(bpy)_3_(PF_6_)_2_ as the photosensitizer (PS) and aqueous HBF_4_ as the proton source. Turnover numbers of 56, 174 and 47 moles of H_2_ per moles of PS were observed for complexes 12–14, respectively. Thus, the present work serves a dual purpose of easy synthesis of functionalized qtpy complexes 12–14 and their use as photocatalysts for HER.

## Experimental section

### General notes and procedures for the synthesis of complexes 12–14

6,6′-(2,2′-bipyridine-6,6′-diyl)bis(1,3,5-triazine-2,4-diamine) 6 was synthesized according to the reported method.^40^ Their complexes with Co(ii), Ni(ii) and Cu(ii), respectively, were prepared by the experimental procedure described below. Other chemicals were commercially available and were purchased and used without any additional purification. Solid of 6 (1.0 equiv., 0.050 g, 0.134 mmol) was added in small portions at 25 °C to stirred solutions of Co(NO_3_)_2_·6H_2_O, Ni(NO_3_)_2_·6H_2_O and Cu(NO_3_)_2_·2.5H_2_O (1 equiv.), respectively, in MeOH (25 mL). The mixtures were refluxed for 12 h and the resulting coloured precipitates were cooled to room temperature, filtered, dried and subjected to crystallization by slow diffusion using dimethyl sulfoxide as a solubilizing agent.

#### Synthesis of [6,6′-(2,2′-bipyridine-6,6′-diyl)bis(1,3,5-triazine-2,4-diamine)](nitrato-O)cobalt(ii)

Complex 12 was synthesized and crystallized in 74% yield by the slow diffusion of EtOAc over DMSO solution. FTIR (ATR): 3436, 3338, 3219, 3165, 3099, 3084, 1661, 1617, 1591, 1567, 1525, 1482, 1454, 1423, 1390, 1313, 1276, 1203, 1165, 1077, 1045, 1029, 991, 955, 832, 800, 758, 742, 705, 681, 648, 625, 572 cm^−1^. HRMS (ESI) calcd for [C_16_H_14_CoN_12_NO_3_]^+^*m*/*z* 495.06690, found 495.06600. Anal. calcd for C_16_H_14_CoN_14_O_6_ : C, 34.48; H, 2.53; N, 35.19. Found: C, 34.71; H, 2.46; N, 34.78.

#### Synthesis of [6,6′-(2,2′-bipyridine-6,6′-diyl)bis(1,3,5-triazine-2,4-diamine)](nitrato-O)nickel(ii)

Complex 13 was synthesized and crystallized in 71% yield by the slow diffusion of EtOAc over DMSO solution. FTIR (ATR): 3458, 3381, 3340, 3230, 3185, 3100, 3083, 1668, 1658, 1616, 1586, 1568, 1523, 1481, 1470, 1418, 1389, 1329, 1313, 1284, 1201, 1179, 1164, 1136, 1071, 1044, 1032, 987, 955, 927, 917, 831, 816, 800, 758, 745, 729, 705, 697, 688, 654, 623, 579 cm^−1^. HRMS (ESI) calcd for [C_16_H_14_NiN_12_]^2+^*m*/*z* 216.0403, found 216.0405. Anal. calcd for C_16_H_14_N_14_NiO_6_: C, 34.50; H, 2.53; N, 35.20. Found: C, 34.72; H, 2.50; N, 34.76.

#### Synthesis of [6,6′-(2,2′-bipyridine-6,6′-diyl)bis(1,3,5-triazine-2,4-diamine)](nitrato-O)copper(ii)

Complex 14 was synthesized and crystallized in 79% yield by the slow diffusion of THF over DMSO solution. FTIR (ATR): 3420, 3360, 3316, 3214, 3154, 3085, 3064, 1655, 1629, 1601, 1590, 1574, 1534, 1522, 1513, 1480, 1470, 1417, 1371, 1323, 1288, 1270, 1201, 1163, 1134, 1083, 1074, 1058, 1040, 981, 951, 912, 827, 799, 765, 749, 720, 700, 660 cm^−1^. HRMS (ESI) calcd for [C_16_H_14_CuN_12_]^2+^*m*/*z* 218.5430, found 218.5378. Anal. calcd for C_16_H_14_N_14_CuO_6_: C, 34.20; H, 2.51; N, 34.90. Found: C, 34.28; H, 3.07; N, 33.68.

### Characterization studies of compounds 6 and 12–14

Crystallographic data were collected using a Bruker Venture Metaljet diffractometer with Ga Kα radiation. The structures were solved by intrinsic phasing using SHELXT in OLEX2, and non-hydrogen atoms were refined anisotropically with Least Squares minimization.^57,58^ Hydrogen atoms were treated by first locating them from difference Fourier maps, recalculating their positions using standard values for distances and angles, and then refining them as riding atoms. Microcrystalline powders were analyzed in transmission-mode geometry using a Bruker D8-Discover instrument (*θ*–*θ* geometry) equipped with an XYZ platform and a HI-STAR gas detector. X-rays were generated using a conventional sealed-tube source with a copper anode producing Cu Kα radiation (*λ* = 1.54178 Å). The samples were gently ground and then mounted on a flat Kapton sample holder. The data collection involved acquisition of two different sections with increasing angular position, giving two different 2D frames. These frames were integrated and combined to produce the final 1D powder X-ray diffraction pattern. Calculated powder X-ray diffraction patterns were generated from the structural data in corresponding CIF resulting from single-crystal analyses, the calculation was performed using Mercury software of the Cambridge Crystallographic Data Centre.^59^ A unique value of the FWHM for the diffraction peaks was adjusted in order to get a better match between the resolution of the experimental and the calculated patterns. The determination of the total carbon, hydrogen, nitrogen, and sulphur (C, H, N, S) content in the compounds was performed using EA 1108 Fisons CHNS Element analyzer with the quantitative ‘dynamic flash combustion’ method. UV-visible spectra were recorded on a Cary 5000. The ATR-FTIR spectra were observed with a Nicolet iS 10 Smart FT-IR Spectrometer within 600–4000 cm^−1^. The thermogravimetric analysis was performed using Mettler Toledo TGA1. The samples were studied from 25 to 800 °C with a heating rate of 10 °C min^−1^.

### Electrochemical measurements of compounds 12–14

Electrochemical measurements were performed in pure dimethylformamide purged with argon at room temperature with a BAS SP-50 potentiostat. Glassy carbon electrode was used as a working electrode, the counter electrode was a Pt wire and silver wire was the pseudo-reference electrode. The reference of electrochemical potential was set using 1 mM ferrocene as an internal standard and the values of potentials are reported *vs.* SCE.^60^ The concentrations of samples were 1 mM. Tetrabutylammonium hexafluorophosphate (TBAP) (0.1 M) was used as supporting electrolyte. Cyclic voltammograms were obtained at a scan rate of 100 mV s^−1^ and current amplitude of 100 μA.

### Photocatalytic experiments

A PerkinElmer Clarus-480 gas chromatograph (GC) was used to measure hydrogen gas evolved from the reaction. The assembly of the chromatograph consists of a thermal conductivity detector, a 7 inch HayeSep N 60/80 pre-column, a 9 inch molecular sieve 13 × 45/60 column, a 2 mL injection loop and argon gas as carrier and eluent. DMF was the solvent of choice for our experiments. Three separate solutions of (1) sacrificial donor and proton source, (2) photosensitizer [Ru(bpy)_3_] (PF_6_)_2_ and (3) catalyst were prepared in order to obtain 5 mL of sample solutions in standard 20 mL headspace vials. In DMF, the resulting molar concentration of photocatalytic components were: 1 M for triethanolamine (TEOA), 0.1 M for (HBF_4_), 0.56 M for water, 0.1 mM for the photosensitizer [Ru(bpy)_3_] (PF_6_)_2_ and 1 mM catalyst (12–14) (pH apparent = 8–9). The resulting mixture was placed on a panel of blue LED 10 W center at 445 nm in a thermostatic bath set at 20 °C which was sealed with a rubber septum and two stainless-steel tubes pierced in it. Argon was carried in the first tube at a flow rate of 10 mL min^−1^ (flow rate adjusted with a manual flow controller (Porter, 1000) and referenced with a digital flowmeter (PerkinElmer Flow Mark)). The second stainless steel tube carried the flow to the GC sample loop passing through a 2 mL over flow protection vial and an 8-port stream select valve (VICCI). Timed injections were done by a microprocessor (Arduino Uno) coupled with a custom PC interface. Corresponding to a specific argon flow, H_2_ production rate was calibrated. For calibration of H_2_ production rate at a specific argon flow, a syringe pump (New Era Pump) equipped with a gas-tight syringe (SGE) and a 26s-gauge needle (Hamilton) was used to bubble different rates of pure hydrogen gas into the sample, to a minimum of 0.5 μL per minute. This gave a linear fit for peak area for H_2_*versus* the flow rates of H_2_. For calibration testing, stock cylinders of known concentration of H_2_ in argon replaced the argon flow (inserted at the pre-bubbler, to keep the same vapor matrix). The measured results independent of flow rate (under same pressure) can be easily converted into the rate of hydrogen using eqn (1). The errors associated to the TON and TOF are estimated to be 10%.^61^

1 Rate of production of H_2_ (μL min^−1^) = [H_2_ standard] (ppm) × Ar flow rate (L min^−1^)

## Conflicts of interest

There are no conflicts to declare.

## Supplementary Material

RA-009-C9RA04303A-s001

RA-009-C9RA04303A-s002
