# Supplementary material for: Mimicking 2,2′:6′,2′′:6′′,2′′′-quaterpyridine complexes for the light-driven hydrogen evolution reaction: synthesis, structural, thermal and physicochemical characterizations
Source: RSC Adv. 2019 Sep 6;9(48):28153–64. doi: 10.1039/c9ra04303a (PMC9071053; doi:10.1039/c9ra04303a)
Supplement: RA-009-C9RA04303A-s001 [file RA-009-C9RA04303A-s001.pdf]

Electronic Supplementary Material (ESI) for RSC Advances.
This journal is © The Royal Society of Chemistry 2019

Supplementary Information

Mimicking 2, 2′:6′, 2″:6″, 2‴-Quaterpyridine Complexes for the Light-Driven Hydrogen Evolution Reaction: Synthesis, Structural, Thermal and Physicochemical Characterizations

Sanil Rajak,[a] Olivier Schott,[b] Prabhjyot Kaur,[a] Thierry Maris,[b] Garry S. Hanan[b] and Adam Duong*[a]

[a]*Département de Chimie, Biochimie et physique* and *Institut de Recherche sur l'Hydrogène,* Université du Québec à Trois-Rivières, Trois-Rivières, Québec, G9A 5H7, Canada

[b]*Département de Chimie,* Université de Montréal, Montréal, Québec, H3C 3J7, Canada

**Contents**		**Page**
I.	**Fig. S1** Thermal atomic displacement ellipsoid plot of the structure of **12** grown from DMSO/EtOAc	S3
II.	**Fig. S2** Thermal atomic displacement ellipsoid plot of the structure of **13** grown from DMSO/EtOAc	S3
III.	**Fig. S3** Thermal atomic displacement ellipsoid plot of the structure of **14** grown from DMSO/THF	S4
IV.	**Table S1** Hydrogen-bond geometry (Å, º) in structure of **12**	S4
V.	**Table S2** Hydrogen-bond geometry (Å, º) in structure of **13**	S5
VI.	**Table S3** Hydrogen-bond geometry (Å, º) in structure of **14**	S5
VII.	**Fig. S4** TGA curves of **6** and **12-14**	S6
VIII.	**Fig. S5** IR spectra of **6** and **12-14**	S6
IX.	**Table S4** IR data and assignments of vibrations in ligand **6** and complexes **12-14**	S7
X.	**Fig. S6.** Cyclic voltammetry of **6, 12** and **13** in full scale	S8
XI.	**Table S5** Cyclic voltammetry data of 2, 2′:6′, 2″:6″, 2‴-quaterpyridine complexes reported in literature	S8
XII.	**Fig. S7** Emission spectrum of blue LED	S9
XIII.	**Table S6** Emission maxima and amplitude of LED light	S9
XIV.	**Fig. S8.** Hydrogen evolution reaction of $Co(NO_3)_2$. $6H_2O$, $Ni(NO_3)_2$. $6H_2O$ and $Cu(NO_3)_2$. $2.5H_2O$ (1 mM each) under blue light. (a) TON's and (b) TOF's.	S9
XV.	**Fig. S9.** Photosensitizer based processes in light-driven hydrogen evolution	S10

	reaction	
XVI.	**Fig. S10.** Heterolytic and homolytic mechanisms of hydrogen evolution reaction catalysed by molecular photocatalyst	S11
XVII.	**References**	S11

*To whom correspondence should be addressed. E-mail: adam.duong@uqtr.ca

X-ray Crystallographic data

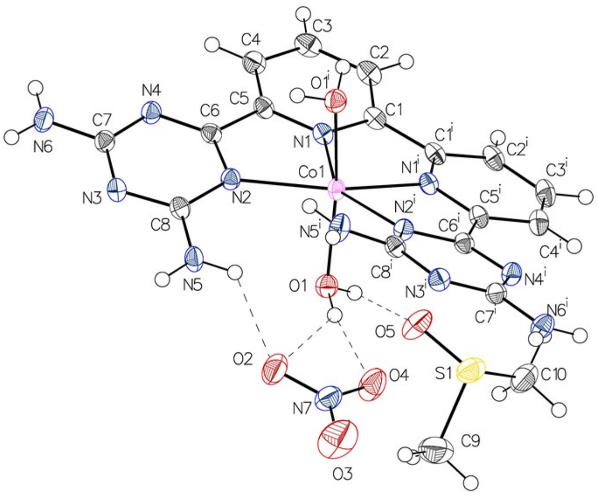

Fig. S1. Thermal atomic displacement ellipsoid plot of the structure of **12** grown from DMSO/ EtOAc. The ellipsoids of non-hydrogen atoms are drawn at 50% probability level, and hydrogen atoms are represented by a sphere of arbitrary size.

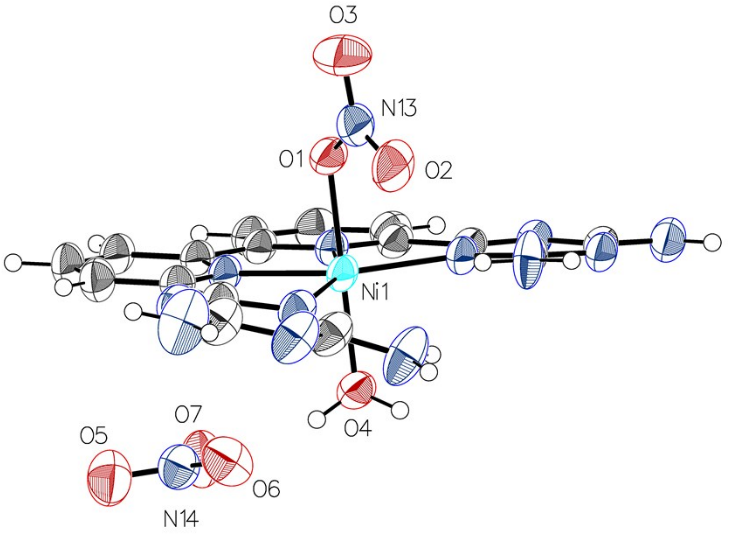

Fig. S2. Thermal atomic displacement ellipsoid plot of the structure of **13** grown from DMSO/ EtOAc. The ellipsoids of non-hydrogen atoms are drawn at 50% probability level, and hydrogen atoms are represented by a sphere of arbitrary size.

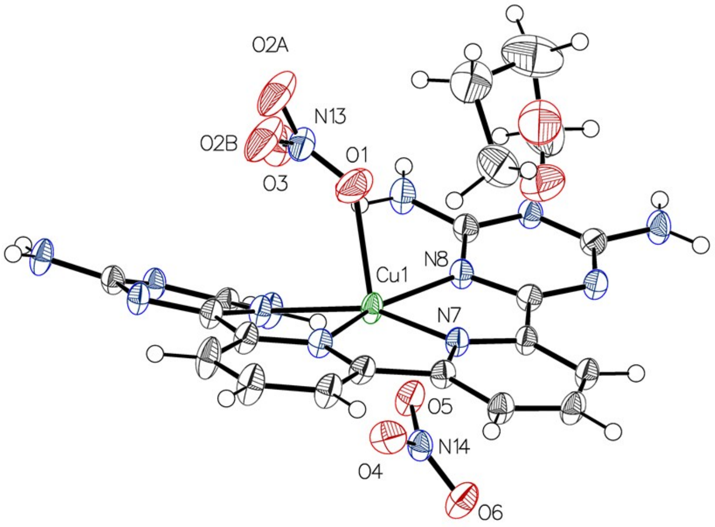

Fig. S3. Thermal atomic displacement ellipsoid plot of the structure of **14** grown from DMSO/THF. The ellipsoids of non-hydrogen atoms are drawn at 50% probability level, and hydrogen atoms are represented by a sphere of arbitrary size

Table S1. Hydrogen-bond geometry (Å, º) in structure of **12**

D—H···*A*	*D*—H	H···*A*	*D*···*A*	*D*—H···*A*
O1—H1*A*···O2	0.88	2.46	3.165 (3)	138
O1—H1*A*···O4	0.88	1.93	2.789 (3)	164
O1—H1*A*···N7	0.88	2.55	3.403 (3)	167
O1—H1*B*···S1	0.88	2.94	3.6667 (18)	142
O1—H1*B*···O5	0.88	1.86	2.727 (3)	167
N5—H5*A*···N3^{i}	0.88	2.24	3.059 (3)	156
N5—H5*B*···O2	0.88	2.16	2.811 (3)	131
N6—H6*A*···O2^{i}	0.88	2.09	2.962 (3)	173
N6—H6*B*···O5^{ii}	0.88	2.03	2.878 (3)	162

Symmetry codes: (i) −*x*+1, −*y*+2, −*z*+1; (ii) −*x*+1, −*y*+1, −*z*+1.

Table S2. Hydrogen-bond geometry (Å, º) in structure of **13**

D—H···*A*	*D*—H	H···*A*	*D*···*A*	*D*—H···*A*
N11—H11*A*···O5	0.83 (4)	2.17 (4)	2.998 (3)	172 (3)
N11—H11*B*···O5^{i}	0.88 (5)	2.59 (4)	3.180 (3)	125 (3)
N11—H11*B*···O6^{i}	0.88 (5)	2.19 (5)	3.051 (3)	165 (4)
N5—H5*A*···O2	0.85 (4)	2.24 (4)	2.957 (3)	142 (3)
N5—H5*A*···N11	0.85 (4)	2.54 (4)	3.153 (3)	129 (3)
N5—H5*B*···O9	0.79 (4)	2.19 (4)	2.954 (4)	161 (4)
N6—H6*A*···O10	0.87 (4)	2.06 (4)	2.926 (3)	173 (3)
N6—H6*B*···O4^{ii}	0.81 (4)	2.25 (4)	3.040 (3)	165 (4)
N12—H12*A*···O8	0.80 (4)	2.11 (4)	2.903 (4)	170 (4)
N12—H12*B*···O7	0.79 (4)	2.23 (4)	2.994 (4)	162 (4)

Symmetry codes: (i) $-x+1$, $-y+1$, $-z+1$; (ii) $-x$, $-y+1$, $-z+1$.

Table S3. Hydrogen-bond geometry (Å, º) in structure of **14**

D—H···*A*	*D*—H	H···*A*	*D*···*A*	*D*—H···*A*
N5—H5*A*···O20*A*^{i}	0.88	2.01	2.822 (7)	153
N5—H5*A*···O20*B*^{i}	0.88	1.97	2.826 (16)	163
N5—H5*B*···O5	0.88	2.14	2.941 (4)	151
N6—H6*A*···O6^{ii}	0.88	2.10	2.966 (4)	169
N6—H6*B*···N9^{iii}	0.88	2.17	3.023 (4)	163
N11—H11*A*···N4^{i}	0.88	2.25	3.129 (4)	176
N11—H11*B*···O3	0.88	2.25	2.972 (5)	140
N11—H11*B*···N5	0.88	2.56	3.130 (5)	124
N12—H12*A*···O4^{iv}	0.88	2.12	2.936 (4)	154
N12—H12*B*···O21*A*^{iv}	0.88	1.96	2.800 (6)	158

Symmetry codes: (i) $-x+1/2, y-1/2, -z+3/2$; (ii) $x-1, y, z$; (iii) $-x+1/2, y+1/2, -z+3/2$; (iv) $-x+3/2, y-1/2, -z+3/2$.

Thermal analysis

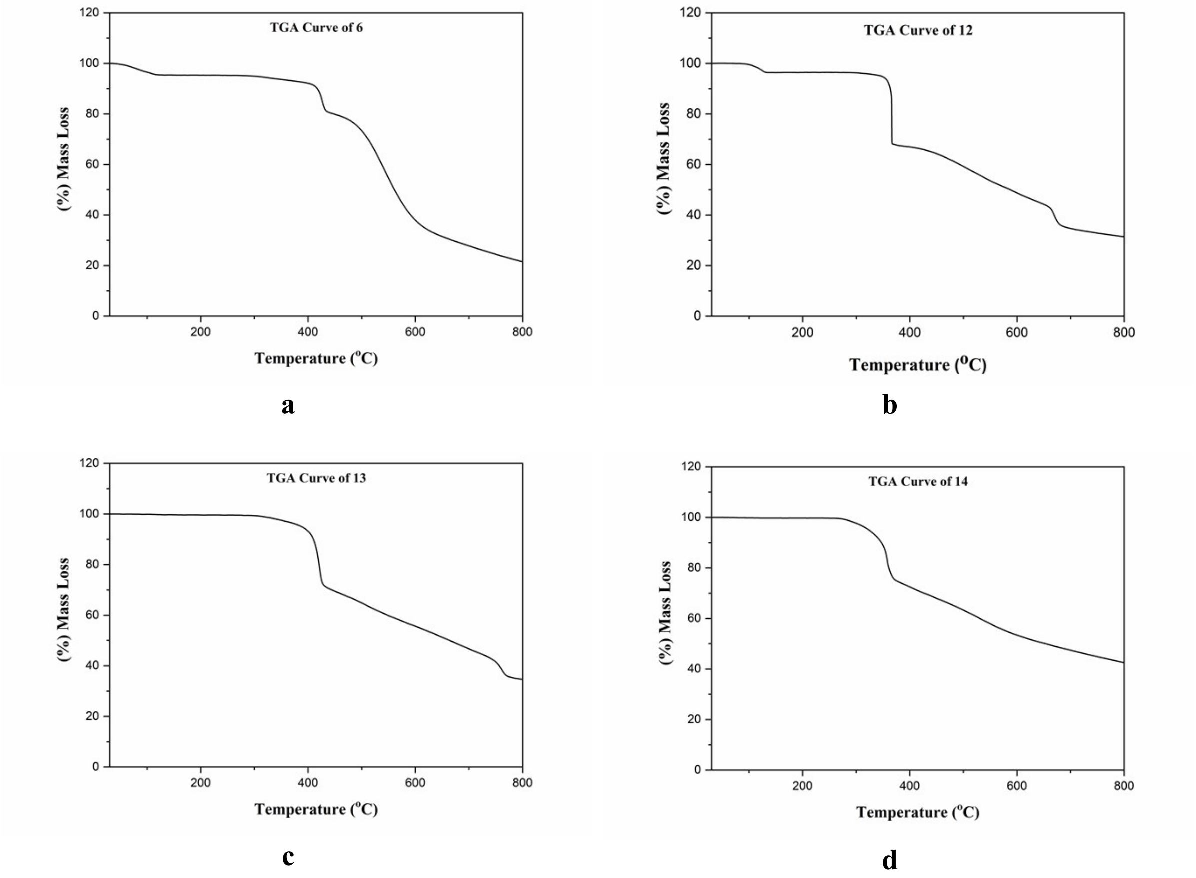

Fig. S4. Thermogravimetric analysis (TGA) curves of **6** and **12-14**

Infrared spectroscopy

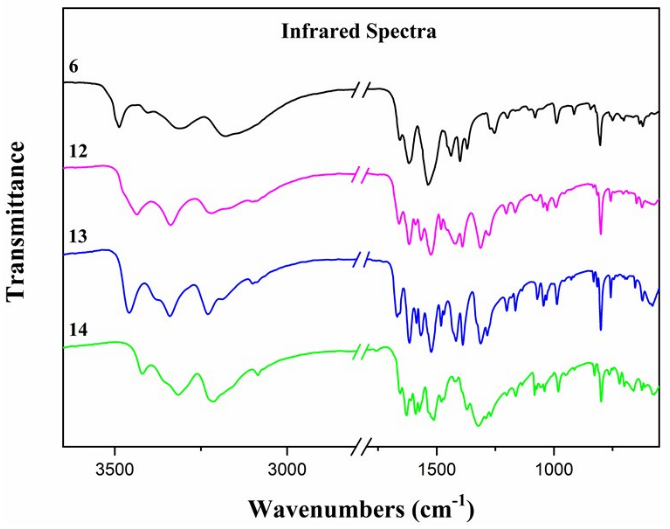

Fig. S5. Infrared spectra of **6** and **12-14**

Table S4. IR data and assignments of vibrations for ligand **6** and complexes **12-14**

$\tilde{\nu}$/cm^{-1}				**Assignment*** **
**6**	**12**	**13**	**14**	
594w	572w	579w	611w	τ (NO_3^-)
621w	625w	623w	625w	Triazine as well as bipyridine ring out-of-plane
635w	648w	654w	660w	def. γ(C-N), γ(C-C)
702w	705vw	705vw	700w	Triazine ring out-of-plane def. γ(C-N), ω(NH_2)
711sh		729vw	720w	
750w	742vw	745vw	749w	ω(NH_2)
761sh	758w	758w	765w	
803m	800m	800s	799m	
820sh	817w	816w	827w	Triazine ring out-of-plane def. γ(C-N), ω(C-H)
843w	832w	831w		
914w		917w	912w	
		927w		Ring breathing both triazine as well as bipyridine
	955vw	955vw	951w	
989w	991w	987w	981w	
	1029w	1032w		
	1045w	1044w	1040w	
			1058w	τ(NH_2)
	1077w	1071w	1074w	
1080w			1083w	
1108sh		1136w	1134w	Bipyridine ring breathing, ρ(NH_2), ρ(C-H)
1157sh	1165w	1164w	1163w	ω(NH_2), δ(C-H), ρ(NH_2)
1198w	1203w	1201w	1201w	Triazine ring def., τ(NH_2)
1254w	1276m	1284m	1270w	
1275w			1288w	ν(C-N) aromatic amines
	1313s	1313s	1323s	ν(NO_3^-)
1370w	1390s	1388s	1371m	ρ(C-H)
1401m				
1439m	1423s	1418s	1417w	
1456w	1454sh	1470w	1470w	ρ(C-H)
	1482w	1481w	1480w	
1537s	1525s	1523s	1513s	Bipyridine ring breathing ν(C-C), δ(NH_2)
			1522sh	
	1567m	1568m	1574m	δ(NH_2)
	1591w	1588w	1590m	
1618s	1617s	1616s	1609w	Triazine ring breathing ν(C-N), δ(NH_2)
			1629m	
1657w	1661m	1658w	1655w	Triazine ring breathing ν(C-N)
		1668m		
			3066sh	
	3084w	3083w	3085w	ν(C-H)
3137s	3099w	3100w		
3178s	3165sh	3185w	3154sh	ν_s(NH_2)
3312s	3219m	3230s	3214s	ν_{as}(NH_2)
3405w	3338s	3340s	3316s	ν_s(NH_2), ν_{as}(NH_2)
		3381w	3360sh	
3487m	3436m	3458s	3420m	ν_{as}(NH_2)

***Abbreviation used for the type of vibration mode. def.: deformation; δ, γ, ρ, τ, ω: bending vibrations; ν: stretching vibration

Cyclic Voltammetry

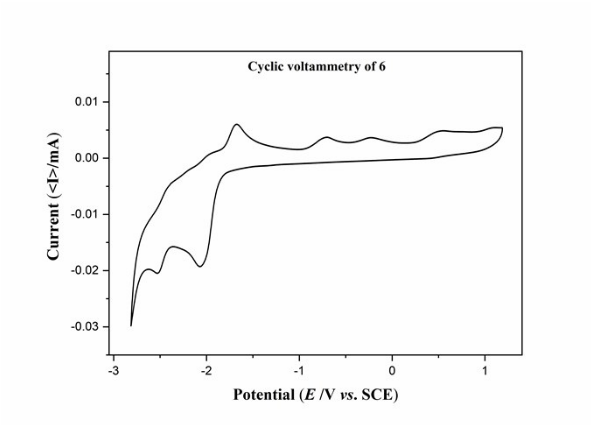

a

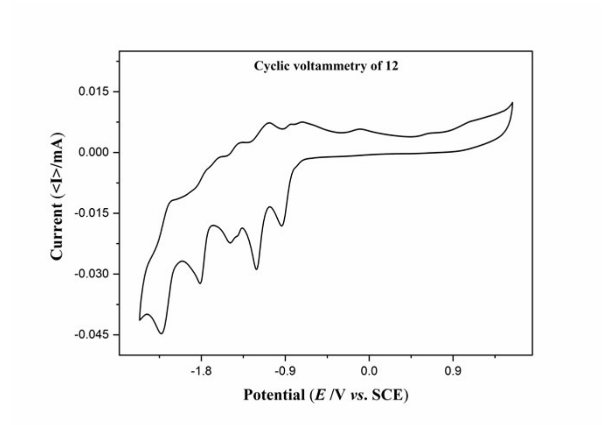

b

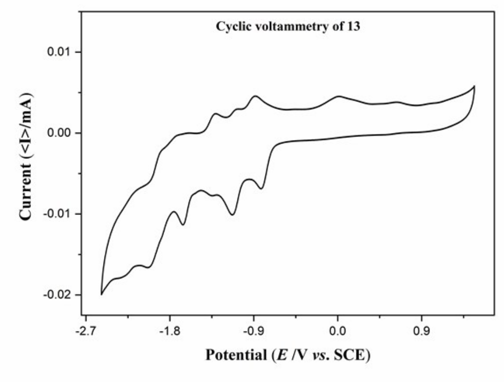

c

Fig. S6. Cyclic voltammetry of **6, 12** and **13** in full scale

Table S5. Cyclic voltammetry data of 2, 2′:6′, 2″:6″, 2‴-quaterpyridine complexes reported in literature

**Compound**	**Solvent**	**Eox1$_{1/2}$ [V]**	**Ered1$_{1/2}$ [V]**	**Ered2$_{1/2}$ [V]**	**Ered3$_{1/2}$ [V]**	**Ered4$_{1/2}$ [V]**
Co(qtpy)$(H_2O)_2(ClO_4)_2$ [1]	MeCN		-0.65	-1.15	---	---
Ni(qtpy)$(ClO_4)_2$ [1]	DMF		-0.79	-1.13	-1.90	---
Cu(qtpy)$(PF_6)_2$ [2]	MeCN	0.13 (nr)	-0.24 (nr)	---	---	---

qtpy = 2, 2′:6′, 2″:6″, 2‴-quaterpyridine, nr = non-reversible

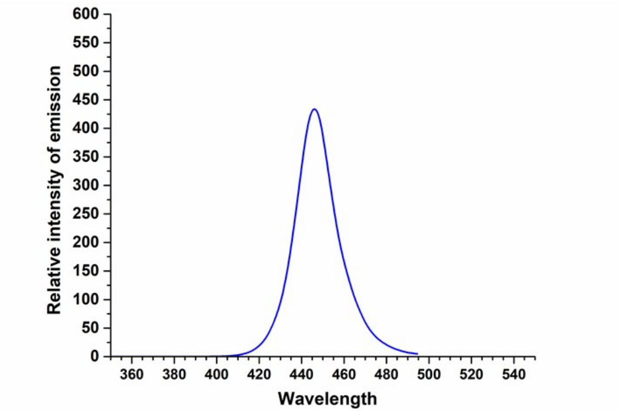

Fig. S7 Emission spectrum of blue LED.

Table S6. Emission maxima and amplitude of LED light.

**Light source**[a]	**Blue**
$\lambda_{max,em}$ (nm)	445
$\Delta\lambda$ (nm)	90
Photon flux in $\mu mol_{photons}.min^{-1}.cm^{-2}$ [b]	20

[a] blue LED 445 nm.

[b] an analog power-meter PM100A (THORLABS) associated with a compact photodiode power head with silicon detector S120C is used to evaluate the photon flux for the LEDs. Photo-diode detector is placed at the same distance from the LED surface than the bottom of illuminated vial.

HER Curves of Co(II), Ni(II) and Cu(II) nitrates

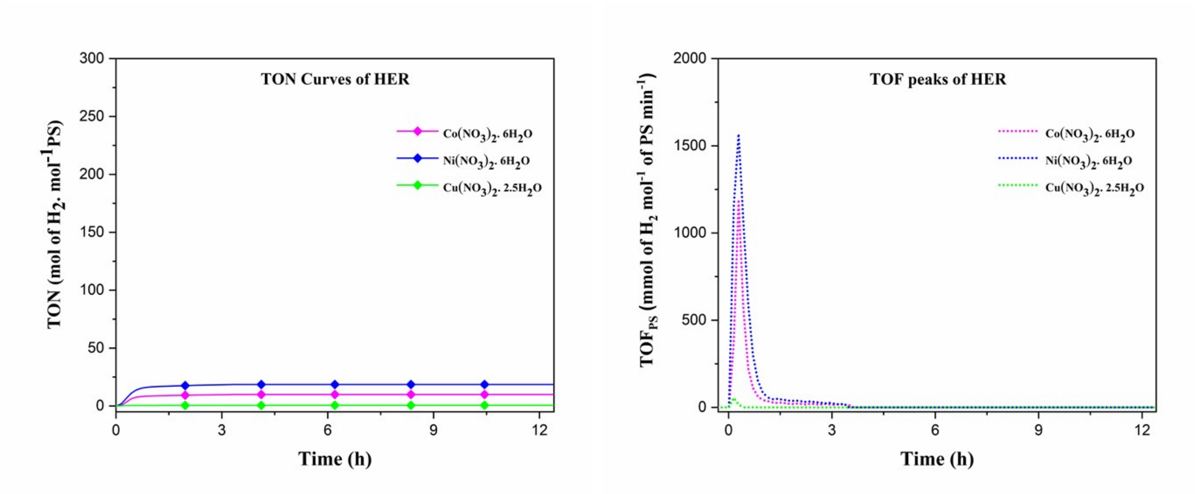

a **b**

Fig. S8. Hydrogen evolution reaction of $Co(NO_3)_2$. $6H_2O$, $Ni(NO_3)_2$. $6H_2O$ and $Cu(NO_3)_2$. $2.5H_2O$ (1 mM each) under blue light. (a) TON's and (b) TOF's.

Mechanism of hydrogen evolution reactions

The mechanism of the hydrogen evolution reaction may occur by two important steps; (I) Activation of the molecular catalyst by the photosensitizer and (II) Redox photocatalytic hydrogen evolution.

Step I

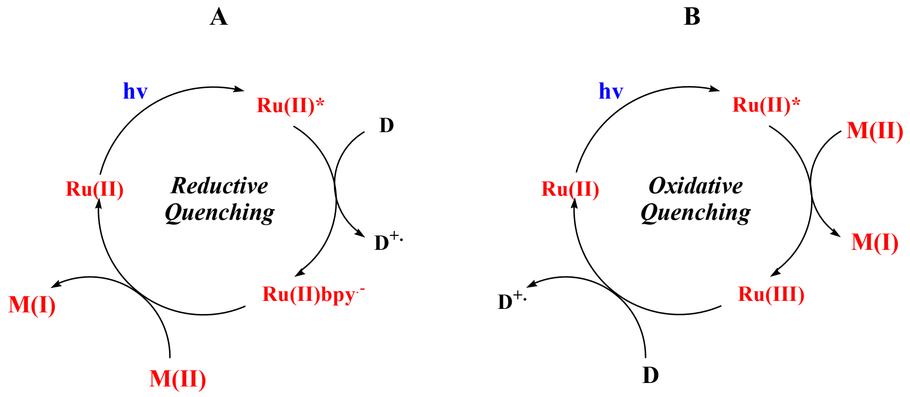

Fig. S9. Photosensitizer based processes in light-driven hydrogen evolution reaction

In step I, activation of the molecular photocatalyst can take place either by reductive or oxidative quenching pathways from the excited photosensitizer (PS*). Visible light is used to for the excitation of the photosensitizer where, in the process of reductive quenching (A), the excited PS* accept an electron from the sacrificial electron donor (SED) and shares it with the molecular catalyst, during this course, the oxidation state of Ru(II) does not change. In the process of oxidative quenching (B), the excited PS* oxidizes and donates its electron to the molecular catalysts and then abstracts an electron from the SED, this process involves redox changes in Ru(II) PS.

In step II, at the photocatalytic centre, the hydrogen evolution can occur by two different mechanisms.

Step II

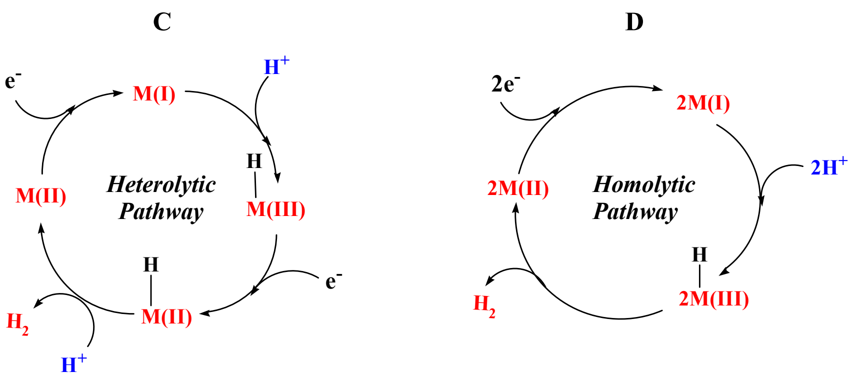

Fig. S10. Heterolytic and homolytic mechanisms of hydrogen evolution reaction catalysed by molecular photocatalyst

In heterolytic mechanism (C), H_2 is evolved by the protonation of the intermediate metal hydride. In the catalytic cycle, the two electrons are transferred either consecutively or alternatively. The H_2 evolution in the alternative homolytic pathway (D) happens by the reductive elimination of two metal hydride intermediates. In both the mechanisms, the metal salts of +II oxidation state undergoes one electron reduction before protonation takes place.[3]

References

1. K-M. Lam, K-Y. Wong, S-M. Yang and C-M. Che, *J. Chem. Soc. Dalton Trans.*, 1995, **7**, 1103-1107.
2. E. C. Constable, S. M. Elder, M. J. Hannon, A. Martin, P. R. Raithby and D. A. Tocher, J. *Chem. Soc. Dalton Trans.*, 1996, **12**, 2423-2433.
3. Artero, V.; Chavarot-Kerlidou, M.; Fontecave, M., *Angew. Chem.* 2011, 50 (**32**), 7238-7266.
